# Consumption of Iodized Salt Among Slum Households of North-East Delhi, India

**DOI:** 10.4103/0970-0218.58406

**Published:** 2009-10

**Authors:** Siddharth Agarwal, Vani Sethi, Deeksha Sharma, Monisha Vaid, Ayushi Agnihotri, Aastha Sindhwani, Pradeep Patra

**Affiliations:** Urban Health Resource Center (UHRC), B-7/122A, Safdarjung Enclave, New Delhi - 110 029, India. E-mail: siddharth@uhrc.in

Sir,

We conducted a household-based cross-sectional study in a purposively selected slum (population: 70 000) of North-East Delhi in August, 2008. The respondents comprised 230 adult females who were involved in cooking food. The sample size of 230 was derived using the following parameters: 50% prevalence, 95% confidence interval, ± 10% confidence interval width, design effect of one, and 5% non-response rate. As no prior estimates of levels of consumption of iodized salt in the studied slum were available, we assumed 50% prevalence. The slum, being large and heterogeneous, was divided into 10 strata. The 10 strata were also the 10 catchment areas of the ASHA workers. Twenty-three respondents were identified in each strata using systematic random sampling methodology.

Using a pre-tested interview schedule, the following aspects were enquired from the respondents - background characteristics, type of cooking salt that they predominantly use; reasons for the same and awareness about the benefits of iodized salt. Iodine content of the cooking salt consumed was tested using a rapid iodine field-testing kit using similar standard procedures, used in the Third National Family Health Survey.(1)

Most (209) households consumed packed refined salt. Only 21 households consumed unpacked crystalline salt (nonpowdered: 19; powdered: 2). The various reasons reported by households consuming crystalline salt (21) included ‘low cost’ (8), ‘since it tasted better’ (9) and ‘feeling it is pure and uncontaminated unlike refined salt’ (4). The value in parenthesis indicates the number of households. Out of those households consuming refined salt (209), 130 consumed it to save time and avoid the inconvenience of grinding crystalline salt, but opted for cheaper varieties owing to low purchasing power. Other reasons reported for consuming refined salt were as follows: better taste and appearance (45), free flowing (24), and influence of television advertisements (10).

A total of 174 out of 230 households were consuming adequately iodized salt (≥15 ppm iodine) [Table 1]. Awareness about health benefits of iodized salt was extremely poor and seen in only 15 out of 230 respondents. Yet on the positive side, all 15 households who were aware of the benefits of consumption of iodized salt were consuming adequately iodized salt. Consumption of adequately iodized salt was higher among households consuming refined salt as compared to those consuming crystalline salt. However, even among households consuming refined salt (209), 36 households were consuming inadequately/non-iodized salt (<15 ppm iodine). Also, consumption of adequately iodized salt was higher in households where the household head was literate.

**Table 1:** Levels of iodized salt consumption in sampled households

	N	Iodine content n (%)		
		
		15 ppm or more	< 15 ppm	0 ppm
Salt consumed				
Crystalline (powdered or non-powdered)	21	1 (4.8)	3 (14.3)	17(81.0)
Refined packed	209	173 (82.8)	32(15.3)	4 (1.9)
Religion				
Hindu	90	74 (82.2)	11 (12.2)	5 (5.6)
Muslim	140	100 (71.4)	24 (17.1)	16 (11.4)
Years of stay in Delhi city				
< 5	18	12 (66.7)	4 (22.2)	2 (11.1)
5-10	24	18 (75.0)	4 (16.7)	2 (8.3)
11-15	25	15 (60.0)	6 (24.0)	4 (16.0)
> 15	163	129 (79.1)	21(12.9)	13 (8.0)
Literacy of household head				
No	109	72 (66.1)	23 (21.1)	14 (12.8)
Yes	121	102 (84.3)	12 (9.9)	7 (5.8)
Literacy of respondents				
No	142	72(81.8)	10(11.4)	6(6.8)
Yes	88	102(71.8)	25(17.6)	15(106)
Awareness about health benefits of iodized salt among households				
Yes	15	15 (100.0)	-	-
No	215	159 (74.0)	35 (16.3)	21 (9.8)
Total	230	174 (75.7)	35 (15.2)	21 (9.1)

Percentages given in this table parenthesis are row%, ppm=parts per million

Three important findings emerged from this study. Although three-fourth households of a north-east Delhi slum were consuming adequately iodized salt, the level of awareness regarding the benefits of consuming iodized salt among the studied population was extremely low. Even among those households consuming refined salt, nearly one-fifth households were not consuming adequately iodized salt. Studies carried out between 2002 and 2004 in Delhi have shown that 64-66% households consume iodized salt.(2,3) Thus, more pursuant efforts need to be made to generate awareness about the health benefits of iodized salt and to enhance demand and availability of iodized salt. Continued dialogue by state-level Iodine Deficiency Disorders (IDD) Control Cell with salt producers and traders and their periodic monitoring would increase production and market availability of adequately iodized salt. Regular community-based awareness activities on the benefits of iodized salt can be conducted through Anganwadi workers, auxillary nurse midwives, nongovernment organizations, self-help groups, and schools. Iodized salt is available at subsidized rate through Targeted Public Distribution System in Chattisgarh, Tamil Nadu, Gujarat, Rajasthan, Andhra Pradesh, Himachal Pradesh, Arunachal Pradesh, Sikkim, Tripura, and Karnataka.(4) Delhi State Government could consider this option to increase the access of iodized salt to the slum population of Delhi.

## References

[1] International Institute of Population Sciences and Macro International (2007). National Family Health Survey-3, 2005-06: India.

[2] Kapil U, Sethi V, Goindi G, Pathak P, Singh P (2004). Elimination of iodine deficiency disorders in Delhi. Indian J Pediatr.

[3] Singh A (2002). Urinary iodine excretion patterns among pregnant women from urban slums of Faridabad - a study. [M.Sc. Thesis].

[4] Atego EA, Sankar R, Schultink W (2005). Pushing in the right direction-steady progress in the control of IDD in India. IDD newsletter.

